# Mapping *Plasmodium falciparum* mutations in Africa: A critical review of emerging drug resistance and implications for malaria control

**DOI:** 10.1016/j.ijid.2025.108033

**Published:** 2025-10

**Authors:** Pierre Gashema, James Kagame, Patrick Gad Iradukunda, Emmanuel Edwar Siddig, Sofonias Kifle Tessema, Merawi Aragaw Tegegne, Mazyanga Lucy Mazaba, Mosoka Fallah, Daniel Ngamije, Jean de Dieu Harelimana, Claude Mambo Muvunyi

**Affiliations:** 1 Africa Centres for Disease Control and Prevention (Africa CDC), Addis Ababa, Ethiopia; 2 Repolicy research Centre, Kigali, Rwanda; 3 Rwanda Biomedical Centre, Kigali, Rwanda; 4 Rwanda Food Drugs and Authority, Kigali, Rwanda; 5 World Health Organization, Geneva, Switzerland

**Keywords:** *Plasmodium falciparum*, Mutations, Resistance, Artemisinin, Africa

## Abstract

Malaria remains a significant public health concern in Africa, with the efficacy of artemisinin-based combination therapies being threatened by the emergence of *Plasmodium falciparum* (Pf) *kelch13* mutations. The current genetic diversity of Pf and associated contributing factors reported in Africa between 2019 and 2024 were reviewed. It was shown that validated *kelch13* mutations are mainly distributed in east African regions, particularly, in Eritrea (R622I, 68%) and Uganda (R561H, 52%). Emerging Pf mutations have been documented in Rwanda (C469F, 36%), Uganda (C469F, 59% and P441L, 69%), and Tanzania (P441L, 20%). Resistance markers for partner drugs, including *Pfcrt, Pfmdr1, Pfdhfr*, and *Pfdhps*, were prevalent in eastern (30-38%) and western African regions (44-55%). The key factors contributing to the development of Pf mutations were identified as drug pressure (23-35%), misuse of antimalarial drugs (10-12.5%), and cross-border population movements (10%). This review posits a critical need to refine malaria control programs, crucially reinforcing drug regulations, enhancing community awareness on appropriate treatment practices, and integrating molecular epidemiologic surveillance systems.

## Highlights

• Validated *Plasmodium falciparum* (Pf) kelch13 mutations rising in east Africa contribute to artemisinin resistance.
• Novel Pf resistance mutations C469F and P441L in east Africa may spread to new regions.
• Increasing Pfcrt, Pfmdr1, Pfdhfr, and Pfdhps markers across Africa threaten artemisinin efficacy.
• Stricter drug regulation, awareness, and molecular surveillance are vital for malaria control.

## Introduction

Malaria remains a leading public health threat in Africa, accounting for 94% of global cases and 95% of malaria-related deaths in 2024 [1]. Currently, artemisinin-based combination therapies (ACTs) are the gold-standard antimalarial drugs used in Africa but their efficacy is declining due to the emergence and spread of drug-resistant *Plasmodium falciparum* (*Pf*) strains in Africa [[2], [3], [4], [5]]. Mutations in the *Pf kelch13 (*R561H, A675V, and P574L) have been reported in some part of Africa and appear less frequent and confer lower-level resistance, often masked by partner drugs, and were associated with delayed parasite clearance [6,7], whereas in southeast Asia, alleles including C580Y, Y493H, and R539T are associated with high-grade resistance and widespread ACT treatment failures [8,9]. In addition, the distribution of K13 alleles (K13 wild-type, K13 R561H, and other K13 mutations) have been documented in 11 countries in eastern (Rwanda, Burundi, Tanzania, and Somalia) and western countries of Africa (Gambia, Burkina Faso, and Sierra Leone) during 2011-2019 [7]. The novel drug-resistant strains are emerging in Africa, and, recently, K13 R561H validated mutation was first reported in Rwanda, the first African Nation reporting artemisinin partial resistance [6]. The understanding of the scale of increasing genetic diversity of *Pf* is critical for monitoring antimalarial drug resistance and guiding interventions. However, factors that promote genomic changes of *Pf* and associated drug resistance are scant. Drug resistance may arise through a complex interplay of factors, including genetic mutations in parasites, selective pressure from drug use, and transmission dynamics [10]. The reports showed that the co-occurrence of candidate mutations P441L, C449A, A568G, and C469F and validated resistance mutations C469Y, R561H, P574L, and A675V suggest the emergence from similar selective pressures, and this poses challenges for the management of *Pf*-induced drug resistance [5]. Of note, the transition for validating candidate mutation markers depends on the consistency of phenotypic impacts across diverse populations [11]. In Africa, where malaria transmission intensity varies, understanding the factors driving *Pf* mutations can be essential for effective drug resistance management. If measures are not taken, the current emergence of drug-resistant *Pf* strains in Africa can reverse decades of progress in malaria control [3]. Although *kelch13* mutations have been implicated in drug resistance and several factors, such as reduced *in vitro* potency, therapeutic failure, and indicative of high prevalence, the validation novel mutations need to be further carried out [12]. Furthermore, the correlation between *kelch13* mutation prevalence and treatment failure rates remains unclear, particularly, in high-transmission settings where other factors may confound resistance dynamics [[13], [14], [15]]. Thus, additional technologies, such as cloning mutations through editing targeted genes that demonstrate the loss of antimalarial drug potency, are warranted. The current data suggest that the malaria surveillance systems should integrate genomic and epidemiologic data to effectively control malaria genetic diversity and implicated drug resistance. These interventions may improve the prediction and prevention of drug resistance spread within the region to adapt control measures. This critical review aimed to synthesize current knowledge on malaria drug resistance markers in Africa and associated contributing factors. These findings inform the present and future challenges for malaria management.

## Methods

A critical literature review, guided by Jesson and Lacey’s framework, was conducted [16]. This robust method allows the comprehensive identification and synthesis of key scientific knowledge on trends, which can guide future research and policy revisions. The statistics reported in this review only considered the countries that have documented *Pf* mutations and potential associated factors.

### Study selection methods

PubMed and Google Scholar databases were systematically searched for peer-reviewed articles from Africa, published between January 2019 and December 2024, that report on malaria drug resistance, candidate or validate markers, and factors driving mutation in Africa. The search strategy included the following keywords: “((malaria drugs resistance OR candidate mutations OR validated resistance markers) AND (factors driving mutation OR malaria drug resistance)) AND (Africa)).” We searched for articles (primary research articles, reports, reviews, and opinions pieces). Studies were included if they reported on malaria drug resistance partners drugs, validated and candidate mutations, and factors driving mutations in Africa in the last 5 years. Studies conducted outside of Africa or missing any of the key criteria were excluded. For data abstraction, we used a standardized form to extract the following information from the studies: author, year of publication, country and regions, study design, target population, sample size, Pfk13 (*Pf* kelch 13 [validated and candidate]), Pfcrt (*Pf* chloroquine resistance transporter), Pfmdr1 (*Pf* multidrug resistance one), Pfdhfr (*Pf* dihydrofolate reductase), Pfdhps (*Pf* dihydropteroate synthase), and factors driving mutations. Three independent authors (P.G., J.K., and P.G.I.) searched the articles to be included in the review and ensure the consistency and quality of the findings, and the research team conducted screening according to the inclusion criteria and harmonized the extracted data.

## Results

### Distribution of the findings reported in African regions

A total of 31,515 articles were obtained from the search engine, of which, 130 studies were included in this study. Of the 130 studies, 123 (94.6%) studies were published between 2020 and 2024. More than one-third (53; 41%) of the studies were from western African regions, 63 (48.5%) from eastern African regions, nine (6.9%) from southern African regions, three (2.3%) from central African regions, and two (1.3%) were conducted in western and eastern regions. Details of the studies included are available as supplementary materials (Table S1, Table S2, Table S3, and Table S4).

### Distribution of P. falciparum validated and candidate mutations in African regions

**Figure 1:**
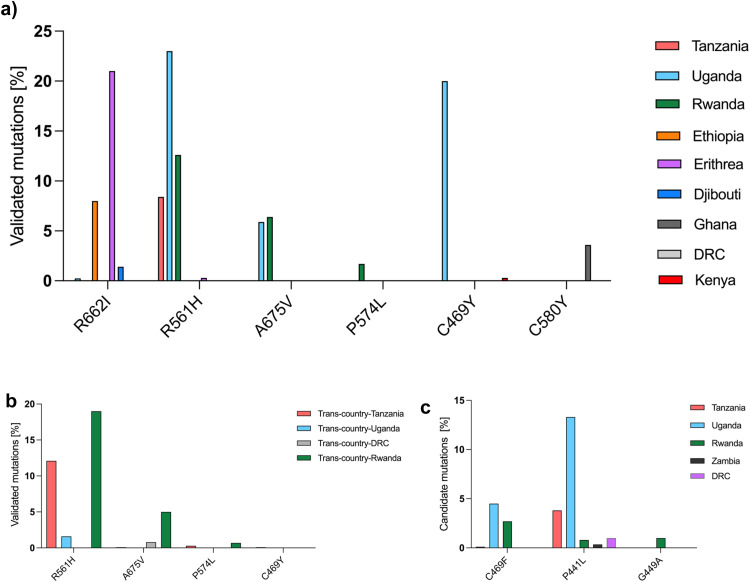
(a, b, and c) Distribution of candidate and validated mutations in African regions.

**Figure 2:**
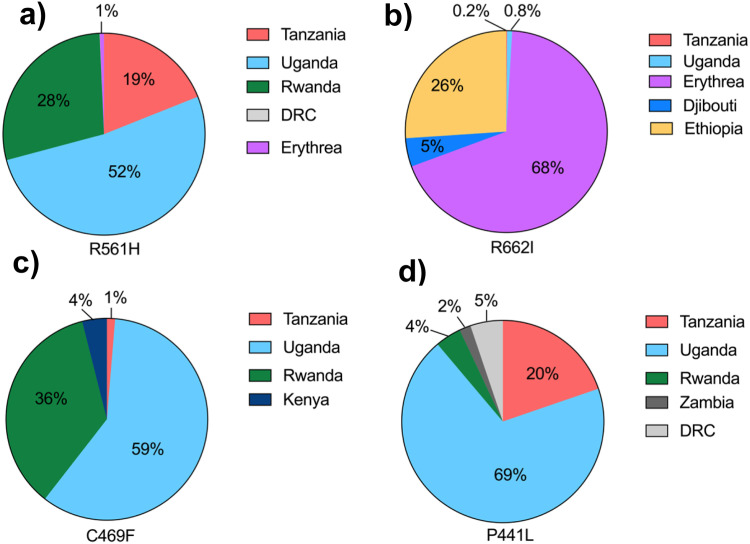
(a, b, c, and d) Frequency of reported candidate and validated mutations in African regions.

*Pf* classic and novel mutations were reported in 23 studies in Africa; 22 of them were primary studies and one was a review study (Table S1). The findings from this review show that R622I, R561H, A675V, P574L, C469Y, and C580Y validated mutations, along with C469F, P441L, and G449A candidate mutations, are variably distributed in African regions. R622I, R561H, C469F, and P441L were frequent in east and central African countries. P574L, C469Y, and C580Y mutations were only recorded in Rwanda, Uganda, and Ghana, respectively. R561H mutations were dominant in multiple countries (Figure 1a, b, c). Considering the countries’ reported *Pf* mutations, the frequency of the validated mutations (R622I) accounted for 68% in Eritrea, 26% in Ethiopia, 5%, in Djibouti, 0.8% in Uganda, and 0.2% in Tanzania. The proportions of R561H mutations were 52% in Uganda, 28% in Rwanda, 19% in Tanzania, and 1% in Eritrea (Figure 2a, b). On the other hand, emerging mutations (C469F) was found in Tanzania (1%), Uganda (62%), and Rwanda (37%); P441L was found in Tanzania (21%), Uganda (73%), Rwanda (4%), and Zambia (2%) (Figure 2c, d).

### Frequency of partner drugs resistance in African regions

The findings from this review highlights the distribution of partner drug resistance in four African regions. Most of the data considered in this review were from the primary studies (Table S2). Overall, partner drug resistance was predominant in eastern and western African regions. The frequency of Pfcrt was 48% in west Africa, 43% in east Africa, 8% in south Africa, and 3% in central Africa. In addition, Pfmdr1 was present at 44% in west Africa, 38% in east Africa, 16% in south Africa, and 2% in central Africa. Moreover, the proportion of Pfdhfr was 48% in west Africa, 36% east Africa, 14% in south Africa, and 2% in central Africa. Lastly, the frequency of Pfdhps was 55% in west Africa, 30%, in east Africa, 14% in south Africa, and 2% in central Africa (Figure S1).

### Frequency of factors driving mutations of P. falciparum in Africa

To comprehensively understand the key drivers of malaria drug resistance, this review included 32 studies conducted in Africa, 31 of which were primary studies (therapeutic efficacy studies, case and observational studies) and one was a review study (Table S3). A total of 17 studies were carried out in west Africa, 12 in east Africa, three in south Africa, and one in western and eastern regions. The most common factors driving mutations in east Africa include drug pressure (23%), insufficient regulation of antimalarial drugs (10%), cross-border movement (10%), drug combinations therapy (ACTs) (8%), and prolonged use of intermittent preventive treatment of malaria during pregnancy with sulphadoxine-pyrimethamine (8%) (Figure S2a). In the western African region, the main contributing factors were drug pressure (35%), transmission intensity (5%), drug resistance selection (5%), epidemiologic factors (5%), and unofficial drug markets (Figure S2b). In southern Africa, drug pressure accounts for 25% of driving factors of mutations and other factors, such as high co-infection rate, drug resistance selection, evolutionary adaptation, drug combination therapy, compensatory mechanisms, and lower antimalarial immunity, accounted for 12.5% (Figure S2c).

### Antimalarial drug resistance and policy implications within the African regions

Finally, we reviewed the policies governing the use of malaria therapies in African regions. Most reported findings were from western Africa, eastern Africa, and central African regions (Table S4). The findings indicated that the efficacy of ACTs varies across African regions, with emerging resistance concerns, particularly, in east Africa, where *Pfkelch13* mutations and partner drug failures are increasingly reported. Although, west and central Africa generally maintain high ACT efficacy with no validated *Pfkelch13* mutations. The emerging resistance markers in Niger and delayed parasite clearance in Rwanda highlight the need for targeted interventions. In east Africa, the spread of artemisinin resistance and the potential decline in partner drug efficacy necessitate adaptive policy responses, including alternative first-line therapies and strengthened surveillance measures. In addition, diagnostic challenges, such as high *HRP2/HRP3* deletions in Eritrea, further complicate malaria control efforts, underscoring the importance of tailored national strategies to sustain treatment efficacy and prevent resistance escalation (Table 1).

**Table 1:** ACT efficacy, resistance, and policy implications within the African regions.

Country	Region	ACT efficacy	Presence of *Pfkelch13* mutations	Resistance concerns	Policy/Control implications
Liberia	West Africa	ASAQ <90%	Not reported	Loss of ASAQ efficacy	NMCP replaced ASAQ with AL.
Togo	West Africa	AL, DP effective	No validated mutations	No ART-R	AL and DP remain first-line.
Sierra Leone	West Africa	High cure rate (ASAQ, AL)	Not reported	High cure rate, but regional risks (e.g. DHA/PPQ failure in SEA)	AL could be a second-line treatment.
Senegal	West Africa	High ACT efficacy	No resistance detected	No ART-R	ACTs remain effective after a decade.
Equatorial Guinea	West Africa	ASAQ, AL highly effective	No resistance markers detected	No ART-R	No immediate concerns.
Niger	West Africa	Emerging SNPs detected	Possible resistance SNPs	Potential challenge to elimination	Strengthen resistance surveillance.
Tanzania	East Africa	High efficacy of ACTs	ART-R detected	Risk to partner drugs due to ART-R exposure	Strengthen surveillance and control measures.
Rwanda	East Africa	AL remains effective but with delayed clearance	*Pfkelch13* R561H confirmed	ART-R spreading, potential risk to partner drugs	Containment strategies needed.
Uganda	East Africa	AL efficacy falling below 90% in some sites	*Pfmdr1* N86 linked to lumefantrine resistance	High recurrence of parasitemia	DP may be considered as first-line.
Eritrea	East Africa	Emergence of *Pfkelch13* 622I	High HRP2/HRP3 deletions	Diagnostic challenges	Adapt malaria rapid diagnostic test strategies.
Chad	Central Africa	ASAQ and AL >98% cure rate	No *Pfkelch13* mutations	No ART-R	No immediate concerns.
DRC	Central Africa	High efficacy of AL	No resistance markers detected	No resistance detected	No policy changes required.

ACT, artemisinin-based combination therapy; AL, artemether–lumefantrine; ART-R, artemisinin partial resistance; ASAQ, artesunate–amodiaquine; DHA/PPQ, dihydroartemisinin/piperaquine; DP, dihydroartemisinin-piperaquine; *Pf, Plasmodium falciparum; SEA, Southeast Asia*.

## Discussion

The present review highlights the current distribution of *Pf K13* mutations across Africa, implicated drug resistance, and suggest mitigation strategies for effective malaria control. The *Pf* validated and candidate mutations have been reported at varying frequencies across the four World Health Organization African regions, with the highest proportions observed in east African regions. This variation in mutations may be attributed to differences in drug pressure, host immune responses in humans and mosquitoes, promoting parasite persistence and transmission intensity, insufficient regulation of antimalarial drugs, and genetic factors [[17], [18], [19]]. Currently, the genetic diversity of *Pf* is significantly increasing in Africa; however, accurate malaria surveillance systems are lacking in several regions, leading to a scarcity of data that truly reflects the situation. This deficiency hinders the development of targeted interventions for effective malaria control. The recent validated R561H mutation, identified in Rwanda [6], has now been reported in all east African countries. In addition, Pf *R622I* and *A675V* mutations were documented in multiple African countries. Uganda reported C469Y and A675, whereas C580Y was noted in Ghana. The candidate *Pf* mutations (P441L, G449A, and C469F) were suggested in Uganda, Rwanda, Democratic Republic of the Congo (DRC), and Tanzania. Moreover, P441L was also described in Zambia, and C469F, P527H, and N537D were recorded in central Africa. Candidate mutations were hardly reported in the west African region (Table S1). The spread of *Pf* mutations in African regions can be linked to multiple independent factors, such as the origins and cross-border transmission, as previously reported [5,17,20,21]. This raises a significant concern for malaria control efforts in endemic regions. Other studies have described the co-occurrence of validated and candidate mutations, suggesting that these mutations may be emerging under similar selective pressure or perhaps as part of the parasite’s adaptive response [5,22]. The observed mutations represent distinct evolutionary trajectories of *Pf* in response to local drug pressure, transmission intensity, and historical treatment policies. For instance, validated *kelch13* mutations are strongly associated with delayed parasite clearance and clinical resistance in southeast Asia and parts of Africa, respectively [7,23], whereas other candidate mutations appear at lower frequencies and may reflect early adaptive responses without yet conferring widespread resistance [24]. The relevance of these mutations lies not only in predicting treatment failure but also in serving as early warning signals for resistance spread. The observed discrepancy between rising molecular markers and sustained therapeutic efficacy reflects the lag between genetic emergence and measurable clinical decline, often masked by long-acting partner drugs and influenced by host immunity and parasite genetics [6,25]. Thus, molecular surveillance and therapeutic efficacy studies are complementary tools. Markers provide advanced signals of shrinking pharmacologic margins, whereas therapeutic efficacy studies confirms clinical outcomes. Recognizing this sequence underscores the need for timely policy adjustments before widespread treatment failures occur. Geographic variations arise from differences in antimalarial drug use (e.g. previous artesunate–amodiaquine vs artemether–lumefantrine deployment), local parasite genetic backgrounds that facilitate or constrain the emergence of resistance, and ecological factors such as transmission intensity and human mobility patterns [[26], [27], [28]]. Understanding the meaning of each mutation within its epidemiologic and pharmacologic context is, therefore, crucial to interpreting the risk of resistance emergence and guiding region-specific control strategies.

This review documents high frequencies of *Pf* drug resistance genes with notable regional variation: Pfcrt (48% west, 43% east), Pfmdr1 (44%, 38%), Pfdhfr (48%, 36%), and Pfdhps (55%, 30%) (Table S2). These patterns reflect differential drug pressure and transmission dynamics across Africa. Historical evidence shows that resistance spreads rapidly once established chloroquine resistance devastated Africa within two decades of emerging from southeast Asia and South America, and dhfr/dhps mutations quickly rendered sulfadoxine–pyrimethamine ineffective [29,30]. More recently, kelch13 mutations emerged independently in Africa after first being reported in southeast Asia [31]. These precedents highlight that molecular markers often signal emerging resistance before clinical failures, underscoring the need for proactive genomics surveillance and timely policy adaptation to avert repeating past public health crises.

This review also highlights that the spread and persistence of antimalarial drug resistance in African regions are driven by multiple factors, including sustained drug pressure, cross-resistance mechanisms, parasite migration, epidemiologic conditions, and health policies. Previous data also reinforced the need for the antimalarial drug resistance and its implications for the World Health Organization global technical strategy [32]. Prolonged use of ACTs, such as artemether–lumefantrine (AL) and artesunate–amodiaquine (ASAQ) antimalarial therapies, along with phased-out drugs, such as chloroquine and Sulfadoxine–pyrimethamine (SP), sustains resistance by selecting for *Pf* mutations, as documented in Tanzania, Mali, Côte d'Ivoire, DRC, Kenya, and Ghana. A previous report suggested changes in malaria treatment policies to deter the spread of artemisinin partial resistance and new antimalarial drug development as a long-lasting solutions to malaria control [33].

This review provides evidence that there are unregulated drugs in some African regions, such as Djibouti, DRC, and Cameroon and this can contribute to sub-optimal drug exposure, fostering incomplete parasite clearance and selection of resistant strains. Similar data were previously noted in Nigeria, suggesting a need to enhance drug regulation policies in Africa [34,35]. Findings of this review pointed out that cross-resistance due to pfmdr1 and *Pfcrt* mutations affect susceptibility to multiple antimalarials in Nigeria and Ghana, whereas reversion to wild-type strains depends on drug withdrawal and genetic factors in Mozambique, Tanzania, and Cameroon. In addition, the movement of resistant parasites across borders, reported between Rwanda, Uganda, Djibouti, and Ethiopia, posit cross-border movements as key contributing factor to ongoing malaria drug resistance. Moreover, as previously reported, parasite and genetic factors of the host are the main drivers to accelerate resistance to antimalarial drugs [36,37].

The findings of this review indicate the regional variations in the efficacy of ACTs across Africa and the emergence of resistance markers, which carry significant implications for malaria control policies. In east African countries, particularly, Tanzania, Eritrea, and Rwanda, face challenges from emerging *PfK13* mutations, highlighting the need to enhance surveillance systems and containment measures [15,20,38]. Data from west Africa indicated a decline in ASAQ efficacy and AL was suggested a substitute. On the other hand, in west and central African regions, ACT drug efficacy remains largely unaffected [39,40], but reduced efficacy was reported in east Africa [13,41], reinforcing target interventions. The widespread distribution of classical and novel *Pf* mutations in Africa underscores the growing threat of treatment failure, highlighting the urgent need for continuous genomics surveillance and tailored intervention strategies to preserve the efficacy of antimalarial therapies.

### Limitations

This review has some limitations. First, data availability across various African regions is inconsistent, with regions such as southern, central, and northern Africa lacking comprehensive studies, which may introduce regional bias. Second, the validation of candidate mutations is still evolving, meaning that some mutations reported in this review may later be reclassified as validated. Despite these limitations, our review offers a comprehensive regional perspective on *PfK13* mutations and drug resistance markers, providing valuable insights to inform malaria control strategies.

## Conclusion

This review highlights predominant classic and emerging *PfK13* mutations and factors contributing to malaria drug resistance in African regions. East African regions showed the highest burden of validated and candidate mutations. Although ACTs remain largely effective, regional disparities warrant tailored intervention strategies, including adaptive treatment policies, strengthened drug regulations, and cross-border collaboration. Enhancing regional systematic surveillance and implementation of operational research will be critical to understanding resistance dynamics and guide control efforts. These measures are essential for preserving ACT efficacy and mitigating the threat of widespread treatment failure in Africa.

## Declaration of competing interest

The authors have no competing interests to declare.

## Appendix Supplementary materials

Image, application 1

## Footnotes

Supplementary material associated with this article can be found, in the online version, at doi:10.1016/j.ijid.2025.108033.
