# Supplementary material for: Mapping Plasmodium falciparum mutations in Africa: A critical review of emerging drug resistance and implications for malaria control
Source: Int J Infect Dis. 2025 Oct;159:None. doi: 10.1016/j.ijid.2025.108033 (PMC12485076; doi:10.1016/j.ijid.2025.108033)
Supplement: Supplementary file 1 [file mmc1.docx]

**Mapping *Plasmodium falciparum* mutations in Africa: A critical review of emerging drug resistance and implications for malaria control.**

Pierre Gashema^1,2^, James Kagame^3^, Patrick Gad Iradukunda^2,4^, Emmanuel Edwar Siddig^1^, Sofonias Kifle Tessema^1^, Merawi Aragaw Tegegne^1^, Mazyanga Lucy Mazaba^1^, Mosoka Fallah^1^, Daniel Ngamije^5^, Jean de Dieu Harelimana^3^, Claude Mambo Muvunyi ^3^*

^1^Africa Centres for Disease Control and Prevention (Africa CDC), Addis Ababa, Ethiopia

^2^Repolicy research Centre, Kigali, Rwanda

^3^Rwanda Biomedical Centre, Kigali, Rwanda

^4^Rwanda Food Drugs and Authority, Kigali, Rwanda

^5^World Health Organization, Geneva, Switzerland

*Corresponding Author: Professor Claude Mambo Muvunyi

Email: [claude.muvunyi@rbc.gov.rw](mailto:claude.muvunyi@rbc.gov.rw). Rwanda Biomedical Centre (RBC).

**Supplementary materials (Figures and Tables)**

**Figure S1:** Frequency of reported markers of partner drugs within African regions


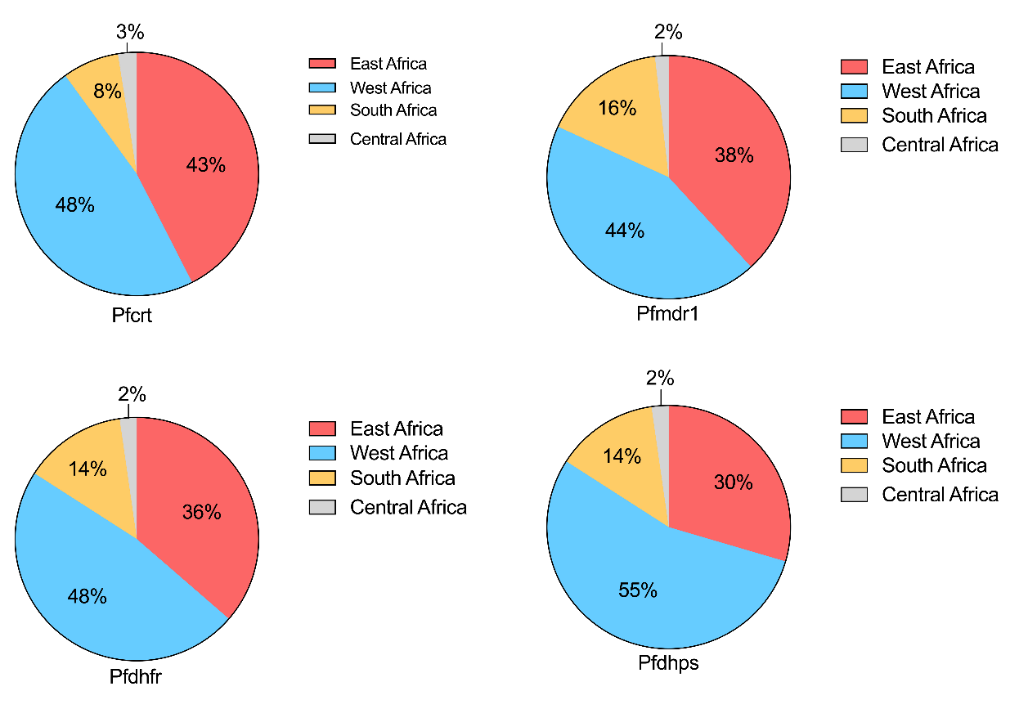


**Figure S2a, b, c:** Frequency of documented factors driving malaria mutations in African regions


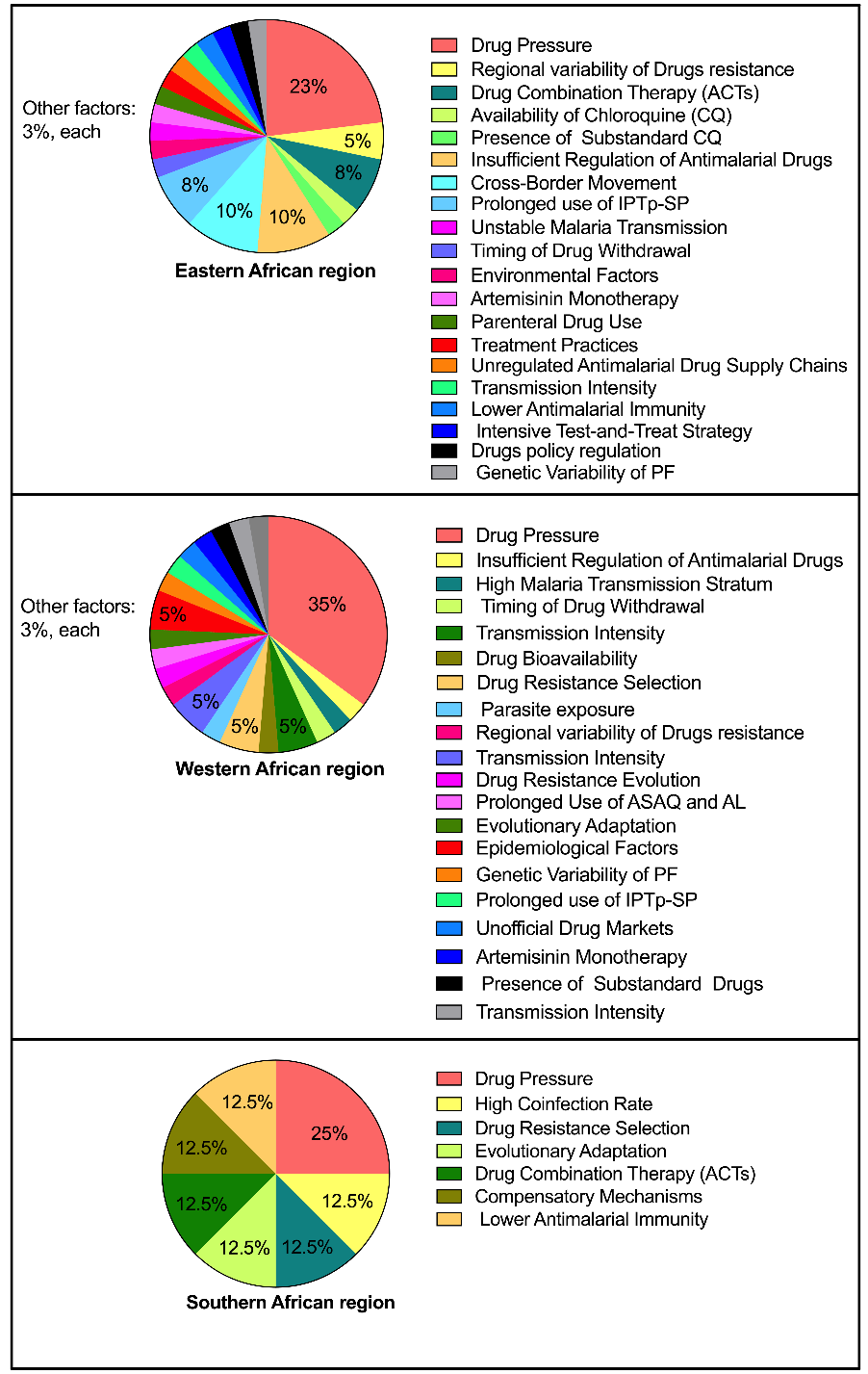


**a)0)**

**c)**

**b)**

| **Table S1 Distribution of Validated and Candidate mutations in African Regions** | | | | | | | | | |
| --- | --- | --- | --- | --- | --- | --- | --- | --- | --- |
|  | **Author, year** | **Year** | **Region** | **Country** | **Validated (%)** | **Candidate (%)** | **Study design** | **Target population** | **Sample size** |
| 1 | Fola A et al | 2023 | East Africa | Ethiopia | R662I  (8) | No | cross-sectional study | Malaria positive cases | 609 |
| 2 | Bakari et al | 2024 |  | Tanzania | R622I (0.05) | No | Therapeutic efficacy studies (TES)(prospective observational study ) | Malaria positive patients | 2015 |
| 3 | Juliano et al | 2023 |  | Tanzania | R561H (7.7), R622I (0.016) | No | Cross sectional study | Samples from Malaria positive patients | 6,278 |
| 4 | Juliano et al | 2024 |  | Tanzania | R561H (1.7) | No | cross-sectional study | malaria positive asymptomatic individuals | 6855 |
| 5 | Moser et al | 2021 |  | Tanzania | R561H (0.23) | No | cross-sectional study | *P. falciparum* clinical isolates | 1,232 |
| 6 | Ishengoma et al | 2024 |  | Tanzania | R561H (24) | No | single-arm, therapeutic efficacy study | P. falciparum positive patients | 343 |
| 7 | (Multicountry) Young et al | 2024 |  | Multicountry (Tanzania) | A675V (0.1), P574L (0.3), R561H (12.1), C469Y (0.1) | C469F (0.1), P441L (3.8) | Cross sectional study | malaria positive cases | 5465 |
| 8 | (Multicountry) Young et al | 2024 |  | Multicountry ( Uganda) | 561H (1.6) | C469F (4.5),  P441L (1.8) | Cross sectional study | malaria positive cases | 5465 |
| 9 | (Multicountry) Young et al | 2024 |  | Multicountry (DRC) | A675V (0.8) | No | Cross sectional study | malaria positive cases | 5465 |
| 10 | (Multicountry) Young et al | 2024 |  | Multicountry (Rwanda) | A675V (5), 574L (0.7), R561H (19) | P441L (0.8), G449A (1), C469F (2.7) | Cross sectional study | malaria positive cases | 5465 |
| 11 | Kahunu | 2024 |  | DRC | R561H (1.4) | P441L (1.0) | Therapeutic efficacy studies (TES)(prospective observational study ) | Malaria positive cases | 179 and 200 |
| 12 | Kayiba et al | 2023 |  | DRC | R561H (0.018) | No | systematic mapping review | Plasmodium falciparum clinical isolates | 6385 |
| 13 | Kirby et al | 2023 |  | Rwanda | R561H (1.34) | No | Cross sectional study | *P. falciparum* positive dried blood spot (DBS) samples | 476 |
| 14 | Uwimana et al | 2021 |  | Rwanda | P574L(1), R561H(13) | No | open-label, single-arm, multicentre, therapeutic efficacy study | Children aged 6-59 months with P falciparum monoinfection | 228 |
| 15 | Makau et al | 2024 |  | Kenya | C469Y (1.3) | NO | Cross sectional study | Malaria positive cases | 226 |
| 16 | Maniga | 2023 |  | Kenya | R539T and R561H | No | Cross sectional study | Symptomatic malaria suspected patients | 231 |
| 17 | Schreidah et al | 2023 |  | Rwanda | R561H (23.5), P574L (2.4),  A675V (6.4), | No | cross-sectional study | Malaria positive patients | 273 |
| 18 | Papa Mze et al | 2024 |  | Djibouti | R622I (1.4) | No | cross-sectional study | P. falciparum positive samples | 79 |
| 19 | Mihreteab et al | 2023 |  | Eritrea | R561H (0.3), R622I (21) | No | retrospective analysis of data from drug-efficacy studies. | uncomplicated malaria patients | 852 |
| 20 | Conrad et al | 2023 |  | Uganda | R561H (23), A675V (9.5) C469Y (13.3) | P441L (23) | longitudinal observational study | Uncomplicated malaria | 150 |
| 21 | Ogwang et al | 2024 |  | Uganda | C469Y (34) | No | retrospective cross-sectional study | Malaria positive samples | 78 |
| 22 | Angwe et al | 2024 |  | Uganda | C469Y (12.5), A675V (2.5) | No | prospective longitudinal study | *P*. *falciparum-*positive patients | 100 |
| 23 | Agaba et al | 2024 |  | Uganda | C469Y (13.5), A675V (5.9) | No | cross-sectional study | Symptomatic malaria suspected patients | 238 |
| 24 | Fola A et al | 2024 | Southern Africa | Zambia | R622I (0.25) | No | retrospective cross-sectional study | *Plasmodium falciparum* positive samples | 389 |
| 25 | Fola A et al | 2024 |  | Zambia | No | P441L (0.35) | cross-sectional study | *P. falciparum* samples collected during the 2018 Zambia National Malaria Indicator Survey | 282 |
| 26 | Aninagyei et al | 2020 | West Africa | Ghana | C580Y (3.6) | No | Cross sectional study | Blood donors | 771 |

| **Table S2 Distribution of markers of partners Drugs** | | | | | | | | | | | |
| --- | --- | --- | --- | --- | --- | --- | --- | --- | --- | --- | --- |
|  |  |  |  |  |  |  |  |  |  |  |  |
| **SN** | **Author** | **Year** | **Region** | **Country** | **Pfcrt** | **Pfmdr1** | **Pfdhfr** | **Pfdhps** | **Study design** | **Target population** | **Sample size** |
| 1 | Papa Mze et al | 2024 | East Africa | Djibouti | Yes | Yes | Yes | Yes | cross-sectional study | P. falciparum positive samples | 79 |
| 2 | Kayiba et al | 2023 |  | DRC | Yes | No | Yes | No | systematic mapping review | Plasmodium falciparum clinical isolates | 6385 |
| 3 | Yobi et al | 2021 |  | DRC | Yes | No | No | No | prospective observational study | patients returning to health centres for fever within 28 days of an initial malaria treatment | 364 |
| 4 | Yobi et al | 2022 |  | DRC | Yes | No | No | No | cross-sectional study | patients with fever or history of fever in the last 24 h | 1087 |
| 5 | Yobi et al | 2021 |  | DRC | Yes | No | No | No | Therapeutic efficacy study | Malaria postive patients | 474 |
| 6 | Kahunu et al | 2024 |  | DRC | Yes | Yes | Yes | yes | Cross sectional study | *. falciparum* populations from patients enrolled in TES | 1,065 |
| 7 | Mukhongo Natabona et al | 2024 |  | Eritrea | No | Yes | Yes | No | Prospective cohort study | P falcioparum positive patients above 20 years | 19 |
| 8 | Fola A et al | 2023 |  | Ethiopia | Yes | Yes | No | No | cross-sectional study | Malaria positive cases | 609 |
| 9 | Osborne et al | 2023 |  | Kenya | Yes | Yes | Yes | Yes | cross-sectional study | asymptomatic and low-density P. falciparum infections from a mass drug administration program | 70 |
| 10 | Zhou et al | 2022 |  | Kenya | Yes | Yes | Yes | Yes | cross-sectional study | Malaria positive samples | 346 |
| 11 | Osoti et al | 2022 |  | Kenya | Yes | Yes | Yes | No | cross-sectional study | *Plasmodium falciparum positive samples* | 300 |
| 12 | young et al | 2024 |  | Multcountry (Rwanda, Uganda, Tanzania and DRC) | No | No | No | No | Cross sectional study | malaria positive cases | 5465 |
| 13 | Uwimana et al | 2021 |  | Rwanda | No | Yes | No | No | open-label, single-arm, multicentre, therapeutic efficacy study | Children aged 6-59 months with P falciparum monoinfection | 228 |
| 14 | Schreidah et al | 2023 |  | Rwanda | Yes | Yes | Yes | Yes | cross-sectional study | Malaria positive patients | 273 |
| 15 | Molina-de la Fuente et al | 2023 |  | South Sudan | Yes | No | Yes | Yes | population-based SMC coverage survey and a longitudinal time series analysis | Malaria positive children (from SMC targeted population) | 532 |
| 16 | Bakari et al | 2024 |  | Tanzania | No | Yes | No | No | Therapeutic efficacy studies (TES)(prospective observational study ) | Malaria positive pateints | 2015 |
| 17 | Juliano et al | 2023 |  | Tanzania | Yes | Yes | Yes | Yes | Cross sectional study | Samples from Malaria positive patients | 6,278 |
| 18 | Juliano et al | 2024 |  | Tanzania | Yes | Yes | Yes | Yes | cross-sectional study | malaria positive asymptomatic individuals | 6855 |
| 19 | Moser et al | 2021 |  | Tanzania | Yes | Yes | Yes | Yes | cross-sectional study | *P. falciparum* clinical isolates | 1,232 |
| 20 | Ishengoma et al | 2024 |  | Tanzania | No | Yes | No | No | single-arm, therapeutic efficacy study | P. falciparum positive patients | 343 |
| 21 | Ishengoma et al | 2019 |  | Tanzania | No | Yes | No | No | single-arm prospective in vivo study | Malaria positive patients | 344 |
| 22 | Sisowath et al | 2009 |  | Tanzania | No | Yes | No | No | Therapeutic efficacy studies (TES)(prospective observational study ) | Malaria positive children | 106 |
| 23 | Conrad et al | 2023 |  | Uganda | No | No | No | No | longitudinal observational study | Uncomplicated malaria | 150 |
| 24 | Ogwang et al | 2024 |  | Uganda | No | Yes | Yes | Yes | retrospective cross-sectional study | Malaria positive samples | 78 |
| 25 | Ebong et al | 2021 |  | Uganda | No | Yes | No | No | randomized, open-label, phase IV clinical trial. | Children aged 6 months to 10 years with uncomplicated falciparum malaria | 599 |
| 26 | Roh et al | 2023 | West Africa | Burkina Faso | Yes | Yes | Yes | Yes | case control study | SMC-eligible children 6-59 months of age diagnosed with malaria | 310 |
| 27 | Nikiema et al | 2024 |  | Burkina Faso | Yes | Yes | Yes | Yes | cross-sectional study | Malaria positive samples | 284 |
| 28 | Tarama et al | 2023 |  | Burkina Faso | Yes | Yes | Yes | Yes | cross-sectional study | lasmodium falciparum-positive cases | 377 |
| 29 | Some et al | 2024 |  | Burkina Faso | Yes | Yes | Yes | Yes | cross-sectional study | uncomplicated malaria patients | 118 |
| 30 | Niba et al | 2021 |  | Cameroon | Yes | Yes | Yes | Yes | systematic review | *studies on SNPs of P. falciparum anti-malarial drug resistance genes from malaria postive samples* | 47382 |
| 31 | Niba et al | 2023 |  | Cameroon | Yes | Yes | Yes | Yes | cross-sectional study | *. falciparum* malaria monoinfections samples | 649 |
| 32 | Nkemngo et al | 2022 |  | Cameroon | No | Yes | No | No | cross-sectional study | Anopheles mosquitoes | 6529 |
| 33 | Nkemngo et al | 2023 |  | Cameroon | No | No | Yes | Yes | cross-sectional study | Adult female Anopheles mosquitoes and Malaria positive pateints | 6529 mosquitoes and 136 malaria positive patients |
| 34 | Mbacham et al | 2023 |  | Cameroon | No | No | No | Yes | cross-sectional study | pregnant women of at least 36 weeks attending ANC | 874 |
| 35 | Tuedom et al | 2021 |  | Cameroon | No | Yes | Yes | yes | cross-sectional study | asymptomatic Plasmodium infection individuals | 2754 |
| 36 | Moukoko Eboumbou et al | 2019 |  | Cameroon | No | No | No | No | prospective hospital-based study | Malaria positive samples | 175 |
| 37 | Niba Ngwa et al | 2023 |  | Cameroon | Yes | Yes | Yes | Yes | cross-sectional study | *P. falciparum*-positive samples | **649** |
| 38 | Kanate-toure et al | 2024 |  | Côte d'Ivoire | No | Yes | No | No | cross-sectional study | asymptomatic and symptomatic malaria patients | 704 |
| 39 | Liu et al | 2022 |  | Equatorial Guinea | Yes | Yes | No | No | retrospective cross-sectional study | Plasmodium falciparum samples | 152 |
| 40 | Boutamba et al | 2023 |  | Gabon | No | No | Yes | yes | cross-sectional study | Malaria positive samples | 70 |
| 41 | Menshah et al | 2020 |  | Ghana | Yes | No | No | yes | cross-sectional study | P. falciparum positive children | 803 |
| 42 | Girgis et al | 2023 |  | Ghana | No | Yes | Yes | Yes | cross-sectional study | P. falciparum positive patients | 196 |
| 43 | Matrevi et al | 2024 |  | Ghana | No | No | Yes | Yes | retrospective cross-sectional study | children aged 9 years and below with uncomplicated malaria | 1,170 |
| 44 | Myers-Hansen et al | 2020 |  | Ghana | Yes | Yes | Yes | Yes | cross-sectional study | P. falciparum isolates from children under five years | 400 |
| 45 | Tornyigah et al | 2020 |  | Ghana | No | No | Yes | Yes | cross-sectional hospital-based survey | Pregnant women attending antenatal clinic | 1957 |
| 46 | Aninagyei et al | 2020 |  | Ghana | Yes | Yes | Yes | Yes | Cross sectional study | Blood donors | 771 |
| 47 | Moss et al | 2023 |  | Guinea-Bissau | Yes | Yes | Yes | Yes | cross-sectional study | Malaria positive samples | 14 |
| 48 | Koko et al | 2022 |  | Liberia | Yes | Yes | Yes | Yes | Therapeutic efficacy studies (TES)(prospective observational study) | Malaria positive children | 359 |
| 49 | Maiga et al | 2021 |  | Mali | Yes | Yes | No | No | Therapeutic efficacy studies (TES)(prospective observational study ) | Malaria positive patients | 337 |
| 50 | Diakite et al | 2019 |  | Mali | Yes | Yes | Yes | Yes | cross-sectional study | Malaria positive cases | 270 |
| 51 | Idowu et al | 2019 |  | Nigeria | Yes | Yes | No | No | cross-sectional study | patients with uncomplicated P. falciparum malaria | 98 |
| 52 | Wotodjo et al | 2023 |  | Senegal | Yes | Yes | No | No | retrospective cross-sectional study | malaria patients and Plasmodium falciparum asymptomatic carriers | 539 |
| 53 | Ndiaye et al | 2024 |  | Senegal | Yes | Yes | No | Yes | retrospective cross-sectional study | *Plasmodium falciparum* samples from febrile patients | 3,284 |
| 54 | Dorkenoo et al | 2024 |  | Togo | No | Yes | Yes | Yes | single arm prospective study | Malaria positive children | 179 |
| 55 | Ebel et al | 2021 | Southern Africa | Angola | Yes | Yes | Yes | Yes | cross-sectional study | Patients with fever, chills , or other malaria symptoms | 50 |
| 56 | Coonahan et al | 2023 |  | Mozambique | No | Yes | Yes | Yes | cross-sectional study | severe malaria patients | 120 |
| 57 | Da silva et al | 2023 |  | Mozambique | No | Yes | Yes | Yes | cross-sectional study | Malaria infected samples | 2251 |
| 58 | Kagoro et al | 2022 |  | South Africa | No | Yes | No | No | sequential mixed-methods study | Malaria positive patients | 4787 |
| 59 | Raman et al | 2019 |  | South Africa | *Yes* | Yes | Yes | yes | cross-sectional study | malaria-positive RDTs | 2393 |
| 60 | Fola A et al | 2024 |  | Zambia | No | Yes | Yes | yes | retrospective cross-sectional study | *Plasmodium falciparum* positive samples | 389 |
| 61 | Fola A et al | 2024 |  | Zambia | No | Yes | No | No | cross-sectional study | *P. falciparum* samples collected during the 2018 Zambia National Malaria Indicator Survey | 282 |
| 62 | Ippolito et al | 2020 |  | Zambia | No | Yes | Yes | Yes | Therapeutic efficacy studies (TES)(prospective observational study) | Maralia positive children | 100 |
| 63 | Issa Souleymane et al | 2023 | Central Africa | Chad | Yes | Yes | Yes | yes | single-arm prospective study assessing the efficacy of AS-AQ and AL | Febrile children aged 6 to 59 months with confirmed uncomplicated P. falciparum | 114 and 101 children were recruited for AL and AS–AQ, respectively. |

| **Table S3. Factors Driving malaria mutations in Africa** | | | | | | | | |
| --- | --- | --- | --- | --- | --- | --- | --- | --- |
| **SN** | **Authors** | **Year of publication** | **Regions** | **Country** | **Key Factors Driving Mutations** | **Study design** | **Target population** | **Sample size** |
| 1 | Ishengoma | 2019 | East Africa | Tanzania | 1: Drug Pressure 2: Regional variability of Drugs resistance | single-arm prospective in vivo study | Malaria positive patients | 344 |
| 2 | Maiga et al | 2021 | West Africa | Mali | 1: Drug Pressure | Therapeutic efficacy studies (TES)(prospective observational study ) | Malaria positive patients | 337 |
| 3 | Yobi et al | 2022 | East Africa | DRC | 4: Availability of Chloroquine (CQ) 5: Presence of Substandard CQ | cross-sectional study | patients with fever or history of fever in the last 24 h | 1087 |
| 4 | Niba et al | 2021 | West Africa | Cameroon | 6: Insufficient Regulation of Antimalarial Drugs 7: High Malaria Transmission Stratum | systematic review | *studies on SNPs of P. falciparum anti-malarial drug resistance genes from malaria postive samples* | 47382 |
| 5 | Moser et al | 2021 | East Africa | Tanzania | 10: Timing of Drug Withdrawal 2: Regional Variability of Drugs resistance 1: Drug pressure | cross-sectional study | *P. falciparum* clinical isolates | 1,232 |
| 6 | Papa Mze et al | 2024 | East Africa | Djibouti | 8: Cross-Border Movement 13: Environmental Factors 6: Insufficient Regulation of Antimalarial Drugs 1: Drug pressure | cross-sectional study | P. falciparum positive samples | 79 |
| 7 | Menshah et al | 2020 | West Africa | Ghana | 10: Timing of Drug Withdrawal 14: Transmission Intensity 15: Drug Bioavailability 12: Drug Resistance Selection | cross-sectional study | P. falciparum positive children | 803 |
| 8 | Girgis et al | 2023 | West Africa | Ghana | 16: Parasite exposure | cross-sectional study | P. falciparum positive patients | 196 |
| 9 | Nkemngo et al | 2022 | West Africa | Cameroon | 1: Drug Pressure 14: Transmission Intensity | cross-sectional study | Anopheles mosquitoes | 6529 |
| 10 | Coonahan et al | 2023 | Southern Africa | Mozambique | 17:Evolutionary Adaptation 3: Drug Combination Therapy (ACTs) 1:Drug Pressure | cross-sectional study | severe malaria patients | 120 |
| 11 | Da silva et al | 2023 | Southern Africa | Mozambique | 18: Compensatory Mechanisms, 19: Lower Antimalarial Immunity | cross-sectional study | Malaria infected samples | 2251 |
| 12 | Conrad et al | 2023 | East Africa | Uganda | 20:Unstable Malaria Transmission 21: Artemisinin Monotherapy 22: Parenteral Drug Use | longitudinal observational study | Uncomplicated malaria | 150 |
| 13 | Matrevi et al | 2024 | West Africa | Ghana | 16: Parasite exposure | cross-sectional study | Symptomatic malaria suspected patients | 238 |
| 14 | Agaba et al | 2024 | East Africa | Uganda | 23: Treatment Practices, 24: Unregulated Antimalarial Drug Supply Chains, 6: Insufficient Regulation of Antimalarial Drugs 1: Drug Pressure | cross-sectional study | Symptomatic malaria suspected patients | 238 |
| 15 | Kanate-toure et al | 2024 | West Africa | Côte d'Ivoire | 25: Drug Resistance Evolution, 26: Prolonged Use of ASAQ and AL | cross-sectional study | asymptomatic and symptomatic malaria patients | 704 |
| 16 | Zhou et al | 2022 | East Africa | Kenya | 1: Drug Pressure 3: Drug Combination Therapy (ACTs) 9: Prolonged use of IPTp-SP | cross-sectional study | Malaria positive samples | 346 |
| 17 | Idowu et al | 2019 | West Africa | Nigeria | 1: Drug Pressure | cross-sectional study | patients with uncomplicated P. falciparum malaria | 98 |
| 18 | Ndiaye | 2024 | West Africa | Senegal | 17:Evolutionary Adaptation 1: Drug Pressure | retrospective cross-sectional study | *Plasmodium falciparum* samples from febrile patients | 3,284 |
| 19 | Fola A et al | 2024 | Southern Africa | Zambia | 1:Drug pressure | retrospective cross-sectional study | *Plasmodium falciparum* positive samples | 389 |
| 20 | Okore et al | 2024 | East Africa | Kenya | 14: Transmission Intensity 19: Lower Antimalarial Immunity | cross-sectional study | *P. falciparum* samples collected during the 2018 Zambia National Malaria Indicator Survey | 282 |
| 21 | Nkemngo et al | 2023 | West Africa | Cameroon | 1: Drug Pressure 12: Drug Resistance Selection | cross-sectional study | Adult female Anopheles mosquitoes and Malaria positive pateints | 6529 mosquitoes and 136 malaria positive patients |
| 22 | Myers-Hansen | 2020 | West Africa | Ghana | 1:Drug pressure | cross-sectional study | P. falciparum isolates from children under five years | 400 |
| 23 | Schreidah et al | 2023 | East Africa | Rwanda | 8: Cross-Border Movement 1:Drug pressure | cross-sectional study | Malaria positive patients | 273 |
| 24 | Nikiema et al | 2024 | West Africa | Burkina Faso | 27: Epidemiological Factors, 1: drug pressure 28: Genetic Variability of PF | cross-sectional study | Malaria positive samples | 284 |
| 25 | Fola A et al | 2023 | East Africa | Ethiopia | 1:Drug pressure 29: Diagnostic and Drug Resistance 30: Intensive Test-and-Treat Strategy | cross-sectional study | Malaria positive cases | 609 |
| 26 | Tarama et al | 2023 | West Africa | Burkina Faso | 27: Epidemiological factors 1: Drug pressure | cross-sectional study | lasmodium falciparum-positive cases | 377 |
| 27 | Tuedom et al | 2021 | West Africa | Cameroon | 1: Drug Pressure 9: Prolonged use of IPTp-SP 31: Unofficial Drug Markets | cross-sectional study | asymptomatic Plasmodium infection individuals | 2754 |
| 28 | Fola A et al | 2024 | West Africa | Zambia | 21: Artemisinin Monotherapy | cross-sectional study | *P. falciparum* samples collected during the 2018 Zambia National Malaria Indicator Survey | 282 |
| 29 | Moukoko Eboumbou et al | 2019 | West Africa | Cameroon | 1: Drug pressure 5: Presence of Substandard Drugs 14: Transmission Intensity | prospective hospital-based study | Malaria positive samples | 175 |
| 30 | Boutamba et al | 2023 | West Africa | Gabon | 1: Drug Pressure 32: Parasite Spread and Transmission | cross-sectional study | Malaria positive samples | 70 |
| 31 | young et al | 2024 | East Africa | Multcountry (Rwanda, Uganda, Tanzania and DRC) | 8: Cross-Border Movement 33: Drugs policy regulation | Cross sectional study | malaria positive cases | 5465 |
| 32 | Yobi et al | 2021 | East Africa | DRC | 1: Drug Pressure 28: Genetic Variability of PF | Therapeutic efficacy study | Malaria postive patients | 474 |

| **Table S4. ACT efficacy, Resistance and Policy implications within the Africa Regions** | | | | | | | |
| --- | --- | --- | --- | --- | --- | --- | --- |
| **SN** | **Authors** | **Year** | **Country** | **Regions** | **Study design** | **Target population** | **Sample size** |
| 1 | Koko et al | 2022 | Liberia | Western Africa | Therapeutic efficacy studies (TES)(prospective observational study) | Malaria positive children | 359 |
| 2 | Dorkenoo et al | 2024 | Togo |  | single arm prospective study | Malaria positive children | 179 |
| 3 | Diallo Alpha et al | 2020 | Senegal |  | randomized, three-arm, open-label study | Malaria suspected patients | 496 |
| 4 | Rivas et al | 2021 | Equatorial Guinea |  | single-arm prospective study evaluating the efficacy of ASAQ and AL | Febrile children aged six months to 10 years with confirmed uncomplicated P. falciparum infection | 226 |
| 5 | Samb Yyade et al | 2023 | Senegal |  | prospective observational study (Ex vivo RSA) | uncomplicated *P. falciparum* malaria | 38 |
| 6 | Arzika Issaet al | 2023 | Niger |  | descriptive and analytical study of *k13* gene polymorphisms using isolates from a randomized, two-armed clinical trial comparing AL versus ASAQ. | . falciparum infected patients with uncomplicated malaria in Niger | 159 |
| 7 | Uwimana et al | 2021 | Rwanda | East Africa | open-label, single-arm, multicentre, therapeutic efficacy study | Children aged 6-59 months with P falciparum monoinfection | 228 |
| 8 | Ishengoma et al | 2024 | Tanzania |  | single-arm, therapeutic efficacy study | *Malaria positive children aged between 6 and 120 months* | 343 |
| 9 | Uwimana et al | 2020 | Rwanda |  | Observational study nested within clinical trials | Malaria positive samples | 340 |
| 10 | Mihreteab et al | 2023 | Eritrea |  | retrospective analysis of data from drug-efficacy studies. | uncomplicated malaria patients | 852 |
| 11 | Ebong et al | 2021 | Uganda |  | randomized, open-label, phase IV clinical trial. | Children aged 6 months to 10 years with uncomplicated falciparum malaria | 599 |
| 12 | Ishengoma et al | 2024 | Tanzania |  | single-arm study on the efficacy of AL and ASAQ | children aged six months to 10 years malaria positive patients | 176 |
| 13 | Issa Souleymane et al | 2023 | Chad | Central Africa | single-arm prospective study assessing the efficacy of AS-AQ and AL | Febrile children aged 6 to 59 months with confirmed uncomplicated P. falciparum | 114 and 101 children were recruited for AL and AS–AQ, respectively. |
| 14 | Yobi Met et al | 2021 | DRC |  | cross-sectional study | isolates of Plasmodium falciparum from treatment failure patients | 364 |
